# Where are the seeds? Lack of floral morphs prevent seed production by the tristylous *Pontederia cordata* in South Africa

**DOI:** 10.1002/ece3.9366

**Published:** 2022-10-01

**Authors:** Sage N. L. Wansell, Sjirk Geerts, Julie A. Coetzee

**Affiliations:** ^1^ Centre for Biological Control, Department of Botany Rhodes University Makhanda South Africa; ^2^ Centre for Invasion Biology, Department of Conservation and Marine Sciences, Cape Peninsula University of Technology Cape Town South Africa

**Keywords:** incompatibility, invasion, management, pollination ecology, *Pontederia cordata*, tristyly

## Abstract

Reproduction is a crucial part of the successful establishment and spread of an invasive species. Invasive plants often produce seeds prolifically to spread into new ranges, yet the invasive macrophyte, *Pontederia cordata* L., does not appear to produce seeds in South Africa, limiting its invasive potential. Here, we aimed to determine what limits seed production of the tristylous *P. cordata* in South Africa, where it is widespread with impacts on the ecology of wetlands it invades, South Africa. We measured floral traits and pollen grain size from populations throughout the invasive range in South Africa to determine the relative proportion of tristylous morphs. We speculated that the absence of specialist native pollinators in the invasive range may be responsible for the absence of sexual reproduction and thus conducted a pollination study to determine whether flowers were visited. Thereafter, we hand pollinated 8865 flowers to conclude whether *P. cordata* exhibited an incompatibility system, which prevented seed production. The floral traits and pollen grain measurements were similar to those reported for short‐morphed flowers from the native range. The pollination study confirmed the absence of specialist insect visitors, while the hand‐pollination experiments resulted in no seed production. Only short‐morphed plants are present in South Africa, and the illegitimate pollination of short‐morphed plants prevents seed production. Vegetative spread through rhizome production is thus responsible for the invasion of *P. cordata* throughout South Africa. These findings suggest that control programs should target the plants' rhizomes to prevent and reduce spread. More importantly, preventing the introduction of medium‐ and long‐morphed plants into South Africa is crucial to preclude *P. cordata* from producing seeds and enhancing invasion.

## Graphical abstract

We aimed to determine what limits seed production of the tristylous Pontederia cordata in South Africa, where it is widespread with impacts on the ecology of wetlands it invades. Floral traits and pollen grain measurements were similar to those reported for short morphed flowers from the native range, and the pollination study confirmed the absence of specialist insect visitors.Only short‐morphed plants are present in South Africa and the illegitimate pollination of short morphed plants prevent seed production.

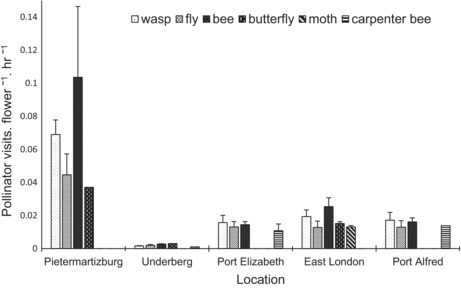

## 1 INTRODUCTION

With about 90% of the world's flowering plants dependent and/or benefiting from animal pollination for seed production (Ollerton et al., 2011), one would expect that pollinator interactions and plant breeding systems for invasive species would be well studied (Le Roux et al., 2010), particularly since plant reproduction is an important barrier for the establishment and invasion of alien plant species (Blackburn et al., 2011). For non‐native plants that rely on sexual reproduction and outcrossing, the pollinators they attract in the novel range could determine their reproduction and thus invasion success or failure (Traveset & Richardson, 2014). Consequently, one would expect a plethora of case studies showcasing how plant reproduction limits plant invasions. There are some well‐known examples, such as *Ficus microcarpa* L. f. (Moraceae), an introduced monoecious fig tree in Florida that was not invasive until its specialized mutualistic wasp pollinator, *Eupristina verticillata* Waterston (Agaonidae), was introduced into the region (Wang, 2014; Zenni & Nuñez, 2013). In areas without *E. verticillata*, *F. microcarpa* remains noninvasive (Van Noort et al., 2013). Similarly, pollination in the alien *Phyla canescens* (Verbenaceae) in Australia by alien honeybees led to seed production and subsequent invasion (Gross et al., 2010). Yet surprisingly few examples highlight failed plant invasions due to a lack of pollinators (but see Bufford & Daehler, 2014), and at best, suggest plant invasions might be slowed down due to pollination limitations (Geerts, 2011; Parker & Haubensak, 2002).

Generalist‐flowered alien plants are assured of pollination when native generalist pollinators are abundant, such as honeybees. Some invasive alien plant genera with generalist flowers that are pollinated by honeybees include *Acacia*, *Banksia*, *Hakea*, *Lythrum*, and *Pueraria* (Geerts & Adedoja, 2021; Geerts et al., 2016; Gibson et al., 2011, 2013; Moodley et al., 2016). Alternatively, if introduced species have specialized pollination systems, they are less likely to receive pollinator services (Adedoja et al., 2021). However, pollinators can adapt, for example, tree tobacco, *Nicotiana glauca* Graham (Solanaceae), is pollinated by hovering hummingbirds (Trochilidae) in its native range in the Americas (Nattero & Cocucci, 2007; Ollerton et al., 2012), while in South Africa, it is pollinated by hovering sunbirds (Nectariniidae). This is surprising since sunbirds have a perching lifestyle and native plants provide them with perches (Anderson et al., 2005), suggesting that a switch to a hovering lifestyle in response to novel resources (*N. glauca* nectar) might be adaptive (Geerts & Pauw, 2009). By contrast, in parts of the novel range, like Greece, where bird pollinators are absent, *N. glauca* has a higher selfing ability (shorter stigma‐to‐anther distances) compared with plants in the native range (Ollerton et al., 2012).

Similarly, large native ranges of pollinators can aid invasion. Formosa lily, *Lilium formosanum* Wallace (Liliaceae), is native to Taiwan but is pollinated by the same long‐tongued convolvulus hawkmoth *Agrius convolvuli* Linn. (Lepidoptera: Sphingidae) in its alien range in South Africa (Rodger et al., 2010). By contrast, other specialized species, such as the moth‐pollinated *Araujia sericifera*, are largely pollinated by generalist native honeybees, *Apis mellifera* Linn. (Hymenoptera: Apidae), in South Africa (Coombs & Peter, 2010). In the absence of specialized pollinators, autonomous self‐fertilization should enhance invasiveness.

Autonomous self‐pollination allows an alien species to overcome pollen limitation and lack of specialized pollinators, thereby escaping negative consequences of mate availability and Allee effects (Baker, 1955; Razanajatovo et al., 2016; Stebbins, 1957; Van Kleunen et al., 2014). Self‐pollination requires a species to have some degree of self‐compatibility to produce seeds for establishment success in an invaded range (Colautti et al., 2010), and it appears that selfing rates are higher in invasive plants than for native plants (Rambuda & Johnson, 2004; Richardson et al., 2000). Similarly, Moodley et al. (2016) found that, although pollinators increased seed set in four out of five invasive Australian Banksia species they studied, all species were capable of autonomous selfing.

The degree of self‐incompatibility in a species may also vary in the native range compared with the invasive range (Colautti et al., 2010; Costa et al., 2017). For example, *Oxalis pes‐caprae* L. (Oxalidaceae), the tristylous Bermuda buttercup weed invading the Mediterranean Basin demonstrated weaker self‐incompatibility in its invasive range than its native range of South Africa due to a strong mate limitation in the invasive range (Costa et al., 2017). Self‐incompatibility is generally a characteristic present in plants that are tristylous, a rare and complex breeding system that ensures optimal seed production and gene flow through cross‐pollination (Ganders, 1979). Each plant in a population possesses only one of three style morphs and all three style morphs may be present in a population (Ornduff, 1966). Tristyly also encourages pollinators to deliver pollen to the correctly matching stigma (Ornduff, 1966). For example, a pollinator would collect pollen from long anthers on a medium‐ or short‐morphed flower and deposit the pollen onto the ‘compatible’ stigma of a long style on a long‐morphed flower. This form of pollination is called ‘legitimate pollination’ and encourages genetic diversity within and among populations (Barrett, 1976).

A complex tristylous breeding scheme frequently comes at a cost. Illegitimate pollination and self‐incompatibility may heavily reduce or prevent fruit and seed production (Barrett & Anderson, 1985). Changes in floral morphologies to promote self‐compatibility are evident in other tristylous species including *Pontederia crassipes* (Martius) [*Eichhornia crassipes* (Martius) Solms‐Laubach] (Pontederiaceae) (Barrett, 1988)*. Pontederia crassipes* is one of the world's most notorious invasive aquatic weeds and has multiple strategies to proliferate, including the loss of a morph to become distylous and the ability to proliferate rapidly via asexual reproduction (Barrett, 1988). However, relying on asexual reproduction can lead to genetic uniformity, which is common in aquatic invaders who disperse with floating vegetative propagules (Barrett et al., 1993; Kliber & Eckert, 2005; Wang et al., 2005). This in turn can lead to the stochastic loss of sexual morphs and the loss of seed production (Hollingsworth & Bailey, 2000; Wang et al., 2005).

Here we address some of these questions by studying the floral morphology, pollinators, and the lack of seeds in the alien range of *Pontederia cordata* L. (Pontederiaceae). In the native range—North, Central, and South America*—*Ornduff (1966) performed legitimate and illegitimate ‘own‐form’ pollination on *P. cordata* in greenhouse experiments and found that illegitimate pollination in all three floral morphologies significantly reduced fruit production. Short‐morphed flowers had the strongest incompatibility with a decline from 61% in legitimately pollinated flowers to only 5% seed set in illegitimately pollinated flowers.

Moreover, there is a distinct difference and separation between *P. cordata* pollen sizes of long‐, medium‐, and short‐morphed anthers, whereby the large anthers possess the largest pollen, the medium anthers possess medium‐sized pollen, and the short anthers produce the smallest pollen (Gettys, 2005). Although *P. cordata* has three different morphs in its native range (Ornduff, 1966), anecdotal field observations throughout its invasive range in South Africa have only recorded populations containing short‐morphed flowers. Thus, it may be possible to match the pollen sizes to the different morphed flowers, because the large and medium‐sized pollen should come from the short‐morphed flowers containing medium and long anthers and so forth (Barrett & Glover, 1985; Gettys, 2005; Price & Barrett, 1982). Short‐morphed flowers in South Africa should therefore have pollen grain sizes of long and medium anthers, similar to that present in the native range (Barrett & Glover, 1985; Gettys, 2005; Price & Barrett, 1982), and may be a characteristic that can help confirm the presence of the short‐morphed plants in South Africa. Furthermore, whether flowers are pollinated by native generalist insects needs to be determined. Specifically, we investigate: (1) whether multiple *P. cordata* floral morphs are present in South Africa; (2) pollen grain sizes of *P. cordata* flowers; (3) pollinators and pollination rates; and (4) seed production and self‐compatibility in *P. cordata*.

## 2 METHODS

### 2.1 Study species

**FIGURE 1:**
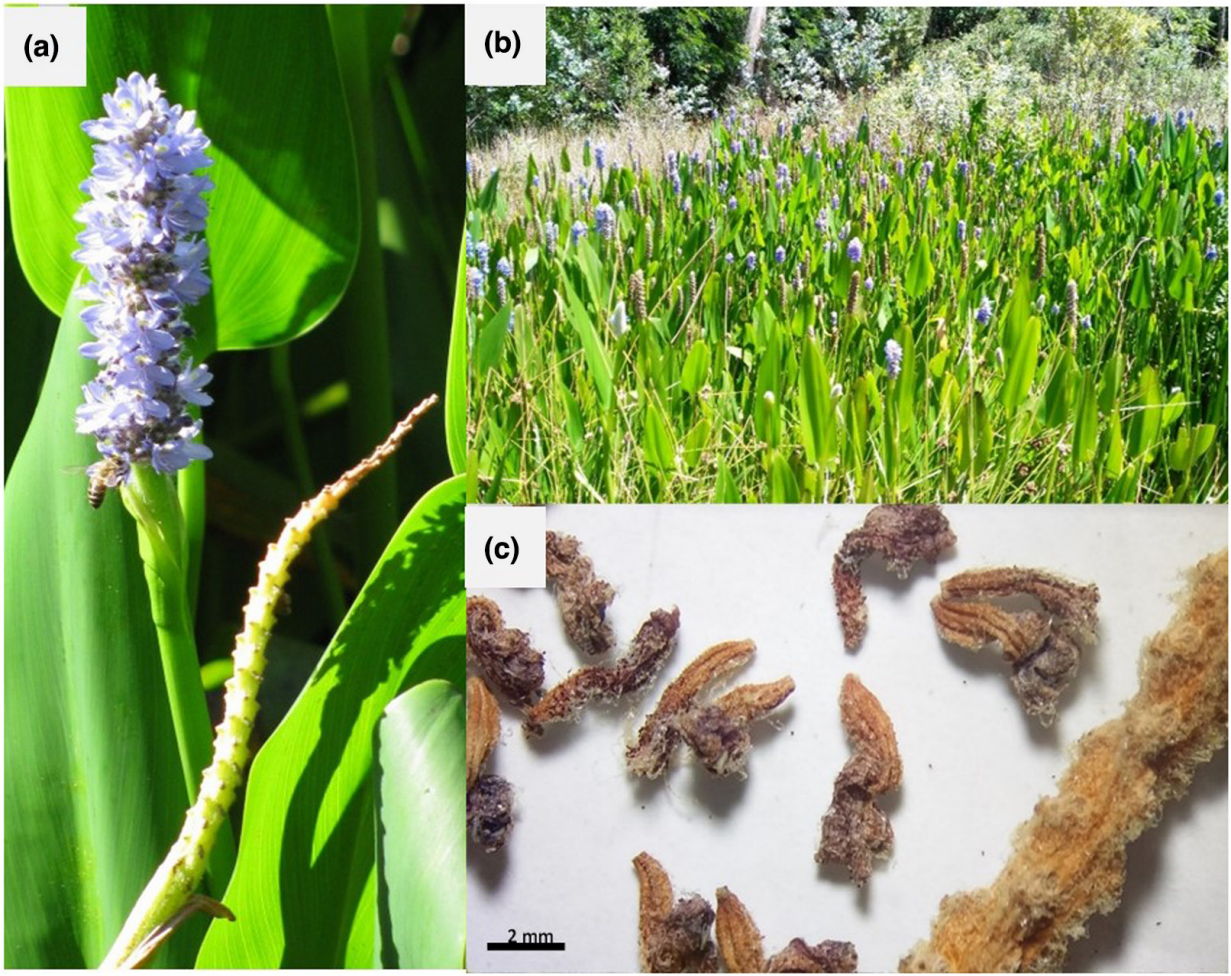
(a) Invasive *Pontederia cordata* inflorescence in bloom with bee pollinator and postbloom with no seed set. (b) Dense *Pontederia cordata* infestation of a wetland at Hogsback Arboretum, South Africa. (c) Dried *Pontederia cordata* flowers approximately 3–4 weeks after artificial cross‐pollination using pollen from a medium anther (photos by S. Wansell, 2019).

Populations of *Pontederia cordata* typically form large colonies in shallow waterbodies such as ponds, streams, wetlands, and riverbanks. Generally, *P. cordata* plants are 1–2 m in height and can be identified by tristylous flowers on a spike that emerges above tall cordate to lanceolate‐shaped green leaves (Figure 1a; Lowden, 1973). *Pontederia cordata* produces blue‐purple flowers and yellow nectar guides on the middle upper lobe (Lowden, 1973; Pellegrini et al., 2018). An individual *P. cordata* flower consists of a short corolla with three upper and three lower lobes spreading outwards, and nectar guides present as two yellow marks (Henderson & Cilliers, 2002). The flower organs generally develop into one style and two sets of three stamens at variable style lengths (Henderson & Cilliers, 2002). It is therefore essential for a pollinator to carry pollen from a different tristylous individual to prevent self‐pollination. Self‐pollinated plants generally produce very few seeds, are more vulnerable to diseases, and tend to have earlier senescence of the flowers' pistil (Scribailo & Barrett, 1994).

Pollinators of *P. cordata* in its native range in eastern Ontario, Canada, include two bumble bees, *Bombus impatiens* Cresson and *Bombus vagans* Smith (Apidae: Bombini), and a specialist bee, *Melissodes apicata* Lovell and Cockerell (Apidae: Anthophorini), that is structurally adapted for pollen collection from *P. cordata* flowers (Harder & Barrett, 1993). In the native range, pollen is collected on different parts of the insect's body and subsequently deposited onto corresponding stigmas of flowers with suitable style morphs, resulting in successful cross‐pollination (Harder & Barrett, 1993).

**FIGURE 2:**
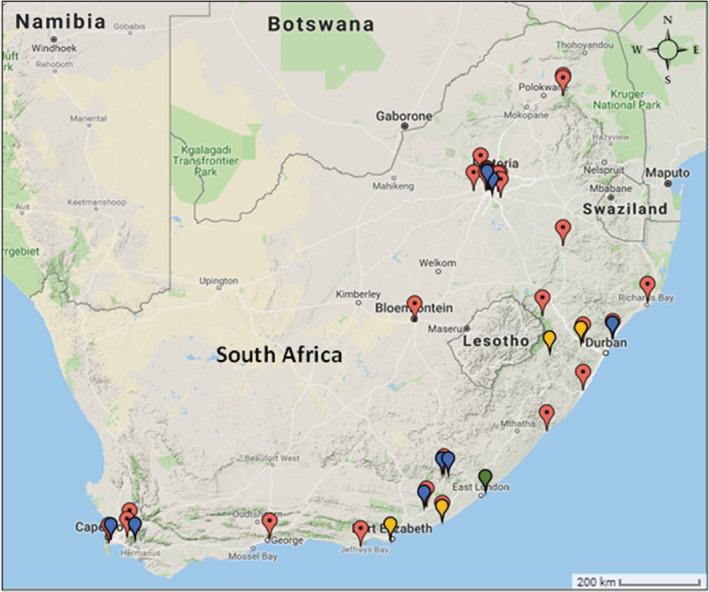
Distribution of invasive *Pontederia cordata* populations in South Africa. Red markers indicate recorded populations (South African National Biodiversity Institute, 2016). Blue markers indicate location of samples collected for flower morphology analysis. Green markers indicate location of samples observed for pollinator visitation analysis. Yellow markers indicate location of samples used for both of abovementioned analyses.

In South Africa, invasive populations of *P. cordata* have been recorded throughout eight of the nine South African provinces (Figure 2). Populations occur in wetlands, waterbodies such as dams, riverbanks, tributaries, and urban environments such as drainage systems and ponds. The long fibrous roots can form dense vegetative mats that block drainage ditches and spread throughout wetlands and irrigated crop fields (Figure 1b; Melton & Sutton, 1991). *Pontederia cordata* populations may also reproduce via clonal growth through rhizome propagation (Melton & Sutton, 1991). Since clonal plants possess the same genetic identities, flowers in a rhizome‐connected population may all possess the same tristylous floral morphology (Ornduff, 1966).

### 2.2 Floral morphology

Flowers from *P. cordata* populations throughout South Africa were measured to determine variation in floral morphology and score floral morphs as short, medium, or long (Table 1). Flower organs included in the floral morphology analysis were stigma, style, filaments, anthers, and petal lengths. At each site, inflorescence samples were collected from a random starting point every 1 m for 10 m to get an average representation of the morphs present in the population. Inflorescence samples were stored in ethanol and distilled water (50% v/v). At each location, 10 inflorescences were analyzed. Measurements of floral organs from 10 random flowers per inflorescence were visualized and captured using WinDIAS 3 Leaf Image Analysis System 3.2. (Delta‐T Devices Ltd., Cambridge, UK). Measurements were recorded in ImageJ (Schindelin et al., 2012). This sampling method was slightly adjusted for Pitermartizburg Botanical Gardens, KwaZulu‐Natal, due to a low sampling size of <10 inflorescence because of invasive plant clearing. All inflorescence was sampled at Pietermaritzburg Botanical Gardens and 10 random flowers per inflorescence were visualized.

**TABLE 1:** Locations, coordinates, and variables studied at each of the *Pontederia cordata* populations in South Africa that were included in this study

Location:	Coordinates	Population size (m^2^)	Number of inflorescences	Floral morphology analysis	Pollen grain size analysis	Pollinator analysis	Artificial pollination experiment
Pietermaritzburg Botanical Gardens, KwaZulu‐Natal	29.6079°S 30.3478°E	~3	<10	x	x	x	
Underberg golf course, KwaZulu‐Natal	29.786865°S 29.491959°E	~858	>10,000	x	x	x	
Durban Botanical Gardens, KwaZulu‐Natal	29.846667°S 31.006667°E	~6	–	x			
Emmarentia Botanical Gardens, Johannesburg, Gauteng	26.161863°E 27.999846°S	~1324	>10,000	x			
Glenhazel, Johannesburg, Gauteng	26.1389°S 28.1020°E	~20	~150	x	x		
Jonkershoek, Western Cape	33.956784°S 18.915221°E	~15	~100	x			
Westlake, Western Cape	34.07560°S 18.45124°E	–	–	x	x		
Port Elizabeth, Eastern Cape	33.9822°S 25.6572°E	~18	~5000	x	x	x	
Royal Port Alfred Golf Course, Eastern Cape	33.600395°S 26.891699°E	~8	~2000	x	x	x	
Gonubie, East London, Eastern Cape	32.936281°S 28.023967°E	~1	~1000			x	
Old golf course in Makhanda (Grahamstown), Eastern Cape	33.296944°S 26.499306°E	–	~1500				x
Hogsback Arboretum, Eastern Cape	32.5952°S 26.9323°E	~308	>8000				x
Hogsback hiking trail, Eastern Cape	32.595056°S 26.94683°E	~6	~3000				x

### 2.3 Pollen grain sizes

The diameter of pollen grains from *P. cordata* populations in South Africa was measured under a scanning electron microscope (SEM) to determine variation in pollen size. Furthermore, since we speculated that only short‐morphed flowers were present in South Africa, these measurements were conducted to compare pollen size with data from the native range. Stigmatic pollen loads were obtained from dried flowers of six *P. cordata* populations (Table 1). Pollen stored in ethanol and distilled water (50% v/v) was prepared for SEM visualization using a gold plating technique according to the developer's manual (Quorum Technologies, 2002). Preparation included placing the pollen and filaments on Double Sided Adhesive Carbon Tape, 8 mm (W) × 20m (L) (Electron Microscopy Sciences, catalog number: 77816) on SME stubs and coating them using a Q150 RS Quorum Rotary‐Pumped Coater using the QT‐Timed Gold vacuum cycle (Quorum Technologies, 2002). Coated samples were analyzed under a TESCAN Vega TS 5136LM SEM using Scadium software (Olympus Scadium software, 2010). Pictures of the equatorial and polar axis of dried pollen grains were captured and measurements were recorded in ImageJ (Schindelin et al., 2012).

### 2.4 Pollinator visitation rates

Field observations of *P. cordata* pollinators in South Africa were conducted on populations across a large part of the invasive range (Table 1; Figure 2). Flowers were observed in the mornings and afternoons for 2 h to ensure all floral visitors were observed as visitation peaks may have differed between pollinator groups. In total, observations for insect visitors took 20 h cumulated across all populations. Pollinator observations were conducted during peak flowering time in spring and summer. Pollinator species, number of flowers visited and number of inflorescences in the population were recorded.

### 2.5 Controlled pollination experiments and effects on seed set

Self‐pollination and cross‐pollination experiments were conducted in a polytunnel greenhouse, using a methodology similar to that of Barrett (1976), to determine the possibility of seed production in invasive *P. cordata* plants in South Africa. *Pontederia cordata* plants (between 40–50) were collected from the old golf course in Makhanda labeled ‘Grahamstown’, Hogsback Arboretum‐labeled ‘Hogsback site 1’, and a Hogsback hiking trail labeled ‘Hogsback site 2’ from the Eastern Cape, South Africa (Table 1). Plants were separated by location and planted in plastic swimming pools (2 m in diameter) in the soil covering the base, with water filled to 10 cm above the soil. Plants were fertilized with Osmocote slow‐release fertilizer (N:P:K = 16:9:12) (Scotts‐Sierra Horticultural Products), at a rate of 5 mg N/L. Plants were watered once a week to ensure water saturated the soil and covered the base of the stems.

Once the plants were budding, fine‐meshed pollinator exclusion bags were placed over each bud. The pollination experiment began as soon as the first flowers on an inflorescence opened. Since short‐morphed flowers were the only morph found in South Africa, all treatments used short‐morphed flowers containing a short pistil and medium and long stamens (Table 2).

**TABLE 2:** Pollination treatments of *Pontederia cordata* flowers from three Eastern Cape populations to test for seed production

Treatment	Total number of treatments per site		
	Grahamstown	Hogsback site 1	Hogsback site 2
Unbagged	436	525	622
Bagged	387	326	511
Self‐pollination: Long anther	377	505	832
Self‐pollination: Medium anther	442	455	680
Cross‐pollination: Short style × Pollen from long anthers of Hogsback site 2	568	–	–
Cross‐pollination: Short style × Pollen from long anthers of Grahamstown	–	444	–
Cross‐pollination: Short style × Pollen from long anthers of Hogsback site 1	–	–	495
Cross‐pollination: Short style × Pollen from medium anthers of Hogsback site 2	462	–	–
Cross‐pollination: Short style × Pollen from medium anthers of Grahamstown	–	415	–
Cross‐pollination: Short style × Pollen from medium anthers of Hogsback site 1	–	–	393

For artificial illegitimate self‐pollination treatments, flowers were marked and emasculated. Pollen was transferred from a flower of a plant from the same location to the stigma of the marked flower, via forceps. Pollinations were made daily on each flower and the number of flowers on each inflorescence was recorded. This process was repeated for 10 inflorescences using pollen from long filaments and 10 inflorescences using pollen from medium filaments.

For artificial cross‐pollination treatments, pollen was transferred from a flower of a plant from a different collection location to the designated stigmas via forceps. Hogsback site 1 anthers were pollinated with pollen from Grahamstown flowers, Hogsback site 2 anthers were pollinated with pollen from Hogsback site 1 flowers, and Grahamstown anthers were pollinated with pollen from Hogsback site 2 (Table 2). This process was repeated for 10 inflorescences using pollen from long filaments and 10 inflorescences using pollen from medium filaments. Unbagged (to include pollinators) and bagged, but untreated controls were also included on 10 inflorescences per control for each population. For all pollination experiments, flowers were observed for 4–6 weeks or until dried flower debris had fallen off the inflorescence to determine whether a seed set had occurred.

To further support the observation of whether seeds were produced or not, a germination experiment on the resultant dried flowers and flower debris was conducted to determine whether seeds were produced. The germination experiment was conducted in a Constant Environment room at 25°C with a lighting setting of 10 h light and 14 h dark. The dried flowers and flower debris were soaked in distilled water and kept damp for 4–5 weeks to encourage germination if any seeds were present (Gettys & Dumroese, 2009).

### 2.6 Statistical analyses

The statistical analysis for the floral organ morphology study used one‐way anovas and Principal Component Analyses (PCA) to determine any differences between the *P. cordata* populations in South Africa for each floral organ. The differences between sites were analyzed in Statistica v.13 (TIBCO Software, 2017). Differences in pollen dimensions between sites were analyzed using the anovas and Tukey's HSD test in Statistica v. 13 (TIBCO Software, 2017). A *t* test was also conducted comparing pollen grain sizes from the invasive range and from short anthers in the native range. Data collected for the pollinator visitation rates were analyzed in Statistica v. 13 (TIBCO Software, 2017) using the one‐way anova and Kruskal–Wallis tests. The Kruskal–Wallis anovas were used to determine differences in the abundance of insect pollinators between populations, irrespective of the plant population sizes recorded during the survey.

## 3 RESULTS

### 3.1 Floral morphology of invasive *Pontederia cordata* plants

**FIGURE 3:**
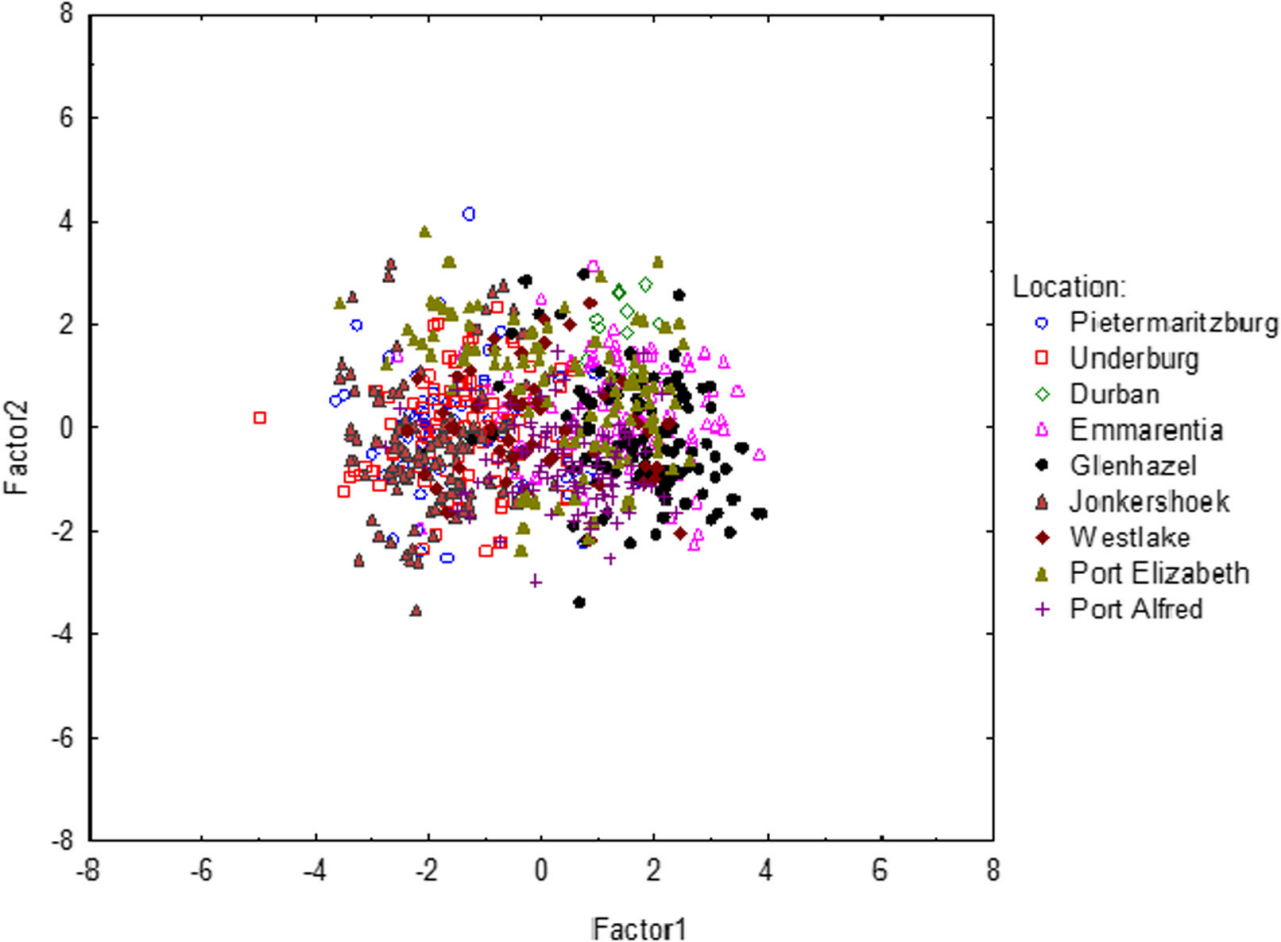
Principle component analysis of floral organs from *Pontederia cordata* populations in South Africa. Floral organs analyzed: Pistil, medium, and long filaments, medium and long anthers, and petal length.

Short‐morphed flowers were the only morph present in South African populations of *P. cordata*. The PCA comparing floral organs clumped variables when analyzing the invasive populations together, and little variability was seen among the different populations (Figure 3). When analyzing each invasive population separately, the floral organs of *P. cordata* plants also exhibited a clumped distribution, with low variability within each population (Figure S1). Factors 1 and 2 made up for 49.56% and 24.27% of the total variation in the analysis, respectively (Figure 3; Table S1).

**FIGURE 4:**
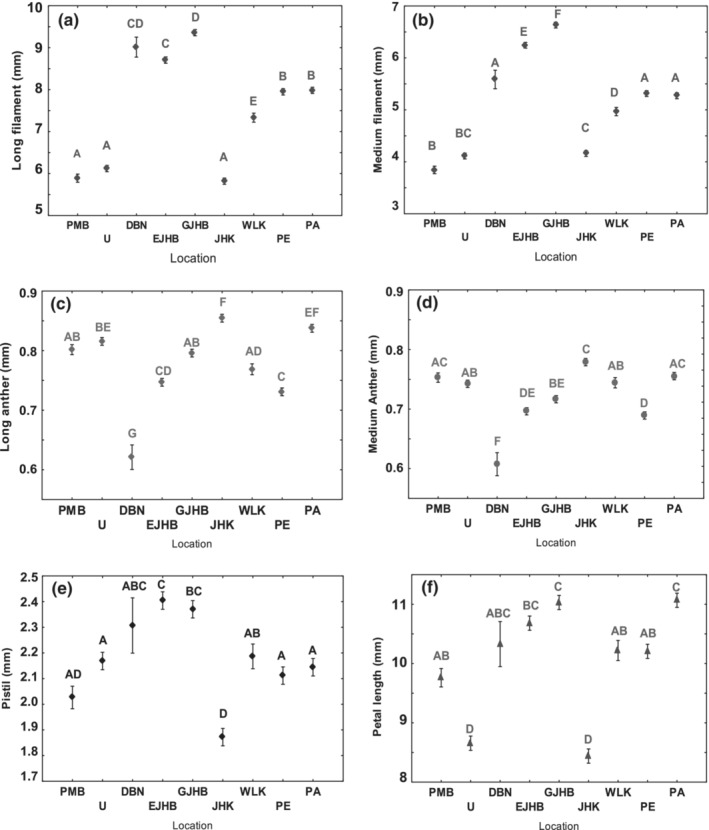
*Pontederia cordata* flower organ measurements from South African populations. Mean values compared by anova; vertical lines denote ± SE bars. The same letters indicate population similarity (Tukey's HSD, *p* < .05). Locations: PMB, Pietermaritzburg; U, Underberg; DBN, Durban; EJHB, Emmarentia; GJHB, Glenhazel; JHK, Jonkershoek; WLK, Westlake; PE, Port Elizabeth; PA, Port Alfred.

Very little variation occurred for each floral organ within a population (Figure 4, Table S2). Pistils were the smallest floral organ measured in every population and varied between 1.85 ± 0.9 and 2.4 ± 0.8 mm (Figure 4e). Pistils were smaller or very similar in comparison to the pistils of short‐morphed flowers of *P. cordata* measured in the United States of America (native region) measuring between 0.8–3.0 mm in height (Table 3). Pistils from invasive populations were also smaller than the medium‐morphed flowers' pistils measuring 5.0–6.0 mm in the native range (Lowden, 1943). Filament lengths recorded in invasive populations were also smaller in size compared with native populations (Table 3). There were no drastic variations in anther lengths (<0.3 mm) between invasive and native ranges for both medium and long anthers (Table 3). Overall, the floral organ sizes of invasive South African populations were very similar to the sizes of floral organs in short‐morphed flowers from the native range (Table 3).

**TABLE 3:** Comparison of floral organ sizes and pollen grain sizes from long and medium anthers of *Pontederia cordata* in South Africa (only short‐morph) to the United States of America for short‐morphed flowers

Floral traits	South Africa (invaded range)	United States of America (native range) for short‐morphed flowers
Pistil length	2.17 ± 0.001 mm	Richards and Barrett (1987): *P. cordata* var. *lanceolata* 2.7 ± 0.1 mm Hazen (1918): 2.7 to 3.0 mm Lowden (1943): *P. cordata var. cordata*: 1.0 mm *P. cordata var. ovalis*: 0.8 mm Lowden (1943) (Medium morph): *P. cordata var. cordata*: 6.0 mm *P. cordata var. ovalis*: 5.0 mm
Filament length	Med: 5.15 ± 0.029 mm Long: 7.1 ± 0.039 mm	Lowden (1943): *P. cordata var. cordata*: Med: 9.2; Long: 14.2 mm *P. cordata var. ovalis*: Med: 6.7; Long: 11.1 mm
Anther length	Med: 0.73 ± 0.02 mm Long: 0.79 ± 0.03 mm	Price and Barrett (1982): Med: 0.85 ± 0.05 mm; Long: 1.02 ± 0.06 mm Lowden (1943): *P. cordata var. cordata*: Med: 0.8; Long: 0.8 mm *P. cordata var. ovalis*: Med: 0.8; Long:0.9 mm
Pollen grain size Fresh (μm)	Med anther pollen: 28.25 ± 0.68 Long anther pollen: 38.50 ± 1.2	Barrett and Glover (1985): Med anther pollen: 53.95 ± 3.6 Long anther pollen: 65.65 ± 3.22 Price and Barrett (1982): Med anther pollen: 46.2 ± 2.0; Long anther pollen: 58.9 ± 7.1
Average pollen grain size (μm)	(50% EtOH): Med anther: 25.40 ± 0.53; Long anther: 34.20 ± 0.52	(Acetolyzed): Gettys (2005): Med anther: 35.04 ± 0.49; Long anther: 44.97 ± 0.3

### 3.2 Pollen grain sizes

The morphology of the pollen grains from invasive *P. cordata* flowers showed a spheroid/biconcave shape but did collapse once gold coated and analyzed under a vacuum (Figure S2). The mean pollen grain size of long and medium anthers in South African populations was 34.20 ± 0.52 and 25.40 ± 0.53 μm, respectively (Figure S3). Pollen grains for both medium and long anthers in South African population were smaller in comparison to North American measurements of *P. cordata* pollen (Table 3). A *t* test comparing pollen grain sizes in South Africa to short anther pollen sizes in the native range indicated no significant differences between them as *p* > .05 (*t* = .15333, *p* = .882).

### 3.3 Pollinator visitation rates

**FIGURE 5:**
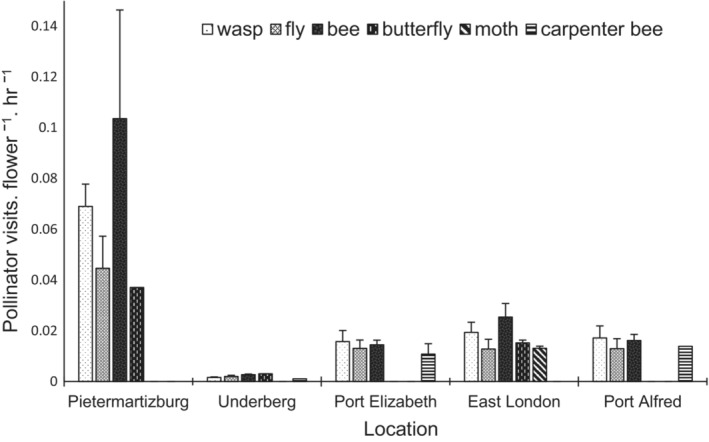
Types of insect pollinators and number of visits on *Pontederia cordata* flowers in South Africa. Vertical lines denote mean ± standard error.

*Pontederia cordata* flowers were visited by Hymenoptera (bees, carpenter bees, and wasps), Lepidoptera (butterflies and moths), and Diptera (flies) species (Figure 5). Underberg and East London had the greatest variety of visiting insects*. Pontederia cordata* flowers in Underberg and East London had visitation rates of 1.40 and 1.23 visits per flower per hour, respectively (Figure 5). Bees, flies, and wasps were the most frequent flower visitors throughout the populations. Pietermaritzburg had the highest abundance of bees visiting flowers (H_3_ = 3.853, *N* = 16, *p* = .2778); however, the abundance of butterflies, flies, and wasps in Pietermaritzburg was approximately the same as the abundance of the insects in the other localities. Overall, there were no significant differences between the different pollinator groups visiting *P. cordata* flowers in every population recorded (Table S3).

### 3.4 Controlled pollination experiments and effects on seed set

No *P. cordata* population in South Africa set any seeds. None of the 2777 cross‐ and 3291 self‐pollination treatments on greenhouse plants produced any seeds (Table 2). Only flower debris was recorded as a result of the pollination experiment (Figure 1c). The subsequent germination experiment of the flower debris to ensure that no seeds were produced resulted in no germination of any of the debris and no seeds were set.

## 4 DISCUSSION

This study shows that *P. cordata* populations in South Africa produce no seeds and only short‐morphed plants are present in the country. We show that there were flower‐visiting insects present among these populations and, even when flowers were artificially pollinated, no seeds were produced. The most common way that invasive plants spread involves both sexual and asexual reproduction. *Pontederia crassipes*, a close relative of *P. cordata* spreads in such a manner (Barrett, 1988; Coetzee et al., 2017). We expected that *P. cordata* invaded South Africa similarly, as propagation via both forms of reproduction would explain why this species has become a highly invasive plant over recent years. However, no sexual reproduction in *P. cordata* was recorded in this study since results from field surveys and pollination experiments showed no seed or fruit production from invasive *P. cordata* plants.

*Pontederia cordata* has an incompatibility system during reproduction to ensure legitimate cross‐pollination and optimal seed production (Ornduff, 1966). Legitimate pollination is present in the native range but does not occur in the invasive range in South Africa. For legitimate pollination to take place, more than one floral morph (short, medium, and long morph) is needed for cross‐pollination (Ornduff, 1966). The first step to determine whether seeds were present in invasive populations in South Africa was to investigate what floral morphs were present. This provided insight as to whether sexual reproduction was taking place among different floral morphs, or whether one or more of these floral morphs were missing.

### 4.1 Floral morphology

Flowers from invasive populations throughout the country were analyzed to determine pistil length (an indicator of the morph of a *P. cordata* plant) and we found that all invasive populations produced flowers with pistils that were indicative of short‐morphed plants. The pistils (<2.5 mm) were smaller than or similar to the pistils of short‐morphed native plants in the United States of America (Hazen, 1918; Richards & Barrett, 1987) except for Lowden (1943) that recorded smaller pistils. Furthermore, other floral organs such as filaments and anthers from invasive South Africa populations were also similar in length to the short‐morphed floral organs in the native range, inferring that the invasive *P. cordata* plants in South Africa are all short‐morphed and sexual reproduction might not occur (Lowden, 1943; Price & Barrett, 1982).

There was little variability in floral organs in invasive plants, suggesting similarity in floral morphology of *P. cordata* throughout South Africa. This suggests that *P. cordata* does not spread via seeds and no cross‐pollination occurs, because low morphological variability in floral traits is generally linked with asexual reproduction, low genetic diversity, and low gene flow (Leles et al., 2015; Zhang et al., 2010). Additionally, the low floral variability present among the invasive populations suggests that populations throughout South Africa may even be clonal populations that are spreading via clonal rhizomes, especially since low genetic variability is present within populations (Wansell, 2021).

The invasive variety in South Africa is not the sterile horticultural variety, ‘Singapore pink’, a cultivar of *P. cordata* that has been traded in aquatic plant nurseries and only propagates clonally (Gettys, 2005), because no pink flowers were observed in any of the field surveys (Wansell, 2021). The slight random variability observed for the different floral organs (Figure 4) could be attributed to phenotypic plasticity—the production of alternative environmentally induced traits by regulatory gene networks in direct response to environmental stresses and heterogeneity, such as variable topography, soil, water‐level and climatic conditions (Pfennig & Ehrenreich, 2014).

### 4.2 Pollen incompatibility

Pollen of invasive *P. cordata* flowers from both long and medium anthers of South African populations was considerably smaller than that of native North American short‐morphed populations, suggesting that they are characteristic of short‐morphed flower pollen, albeit smaller in size than native *P. cordata* pollen. The lack of other morphed plants in the invasive range in South Africa prevented the comparison of pollen from short‐, medium‐, and long‐morphed invasive plants and thus the identification of any pollen trimorphism in the country was not possible. Long anther pollen was, however, larger than medium anther pollen, synonymous to past studies (Barrett & Glover, 1985; Gettys, 2005; Price & Barrett, 1982). Pollen size variability was present among the different invasive populations in South Africa; however, no significant variations were observed and thus all populations shared the same short‐morphed flowers that produce this pollen. It may be possible that the very small, variable pollen grains observed in this study cause a strong incompatibility system and no seed production, or that the pollen itself may be sterile due to abnormalities in pollen development related to continuous vegetative reproduction (Smith, 1898). Bufford and Daehler (2014) studied four noninvasive ornamental plants in Hawai'i and determined that low pollen viability can cause seed production failure and act as a barrier to plant invasion. A study by Barrett & Glover (1985) investigated the trimorphic incompatibility of the tristylous species, *Pontederia sagittata* Presl. (Pontederiaceae), an emergent macrophyte native to Central and South America that was once thought to be a variety of *P. cordata*. Their studies in the native range were similar to this study in the invasive range and included flower and pollen measurements, controlled pollinations, analysis of the behavior of pollen tubes, and field studies in Mexico (Glover & Barrett, 1983). Glover and Barrett (1983) determined that a very strong self‐incompatibility system was present for *P. sagittata*, especially for long‐ and short‐morphed flowers. They found that pollen from long anthers of short‐morphed flowers produced almost no seeds during self‐pollinations because only 9.4% out of 457 short‐morphed flowers self‐pollinated by long anthers produced fruit (Glover & Barrett, 1983). Furthermore, they deduced that illegitimate cross‐pollinations presented cross‐incompatibility and produced similar results to that of self‐pollination (Glover & Barrett, 1983). Similar results were obtained from self‐pollination studies of *Narcissus tazetta* L. (Amaryllidaceae) (Dulberger, 1964) in distylous native populations in Israel, whereby self‐pollinated short‐ or long‐styled plants proved to be almost or entirely sterile during breeding experiments, but cross‐pollinated plants produced fertile seeds. Pollen sterility or low pollen viability may be caused by genetic bottlenecking and deleterious reproductive mutations (Bufford & Daehler, 2014). These deleterious reproductive mutations can act as an invasion barrier by preventing seed production and extensive invasion via sexual propagation (Bufford & Daehler, 2014).

Further studies on the incompatibility system of invasive short‐morphed plants in South Africa should analyze the behavior of pollen tubes from short‐morphed flowers to confirm the types of mechanisms inhibiting fertilization or germination during illegitimate pollination. Furthermore, genetic analyses on the invasive populations should be conducted to determine the genetic diversity, introductory events, and possible genetic bottlenecking in invasive populations (Barrett, 2015; Paterson et al., 2009).

### 4.3 Pollination and insect visitations

We also speculated that insufficient pollination caused by a lack of pollinators or a specialized pollination system could be the cause of no seed production in invasive populations. Although not the only pollinator, a specialist solitary bee pollinator, *Melissodes apicata* Lovell and Cockerel (Anthophoridae), is co‐adapted to tristylous floral polymorphisms present in *P. cordata* populations in the native range of North America (Harder & Barrett, 1993). *Melissodes apicata* has behavioral and morphological adaptations for collecting *P. cordata* pollen and nectar such as possessing proboscides with tiny hairs for collecting the appropriate pollen (Laberge, 1963; Wolfe & Barrett, 1987). Apart from *M. apicata* in the native range in the USA, there are also generalist insects that pollinate *P. cordata*, such as generalist honeybees and bumble bees, *Bombus impatiens* Cresson and *Bombus vagans* Smith (Apidae) (Harder & Barrett, 1993). Despite the lack of *M. apicata* in South Africa, invasive populations were observed being abundantly visited by a large variety of generalist pollinators in this study. The other factor besides the abundance of pollinator visitations is the duration of foraging bouts. Further studies should include the observation of the pollinator visit duration and identification of the pollinators. The presence of generalist insects such as honeybees visiting invasive *P. cordata* flowers is an indication that the lack of seeds is not due to a specialized pollination system nor due to a lack of pollinators.

### 4.4 Artificial hand pollinations

To strengthen our investigation further, 8865 *P. cordata* flowers from invasive populations in South Africa were artificially pollinated in various treatments at optimal growth conditions to determine whether any seeds could be produced. Since no seeds developed from any of these flowers, it was concluded that seeds are not produced in invasive populations in South Africa, most likely due to the self‐incompatibility of flowers from short‐morphed plants. Asexual reproduction is therefore responsible for *P. cordata*'s spread, and it is likely that plants are spreading via rhizomes only. It is therefore imperative that further introduction of *P. cordata* into South Africa is prevented to avoid the possibility of another morph being introduced that could legitimately cross‐pollinate the short‐morphed plants to produce seeds. *Pontederia cordata* produces copious amounts of seeds in the native range that are small, buoyant, and more easily dispersed than rhizomes (Gettys & Dumroese, 2009). Although *P. cordata* is already an invasive macrophyte in South Africa, sexual reproduction would dramatically increase the species invasiveness and allow *P. cordata* to easily spread between waterbodies compared with slower spread by rhizomes.

The current spread of *P. cordata* in South Africa may be perpetuated by avid gardeners and horticulturists through trading and dumping of plants, asexual propagules, and underground fragments in ditches, streams, and other waterbodies. Furthermore, it is highly likely that fish farmers and golf course owners may be utilizing *P. cordata* as a stabilizing plant for dams and waterbodies, ignorant of the species' NEMBA 1b invasive status and threat to the ecosystem (National Environmental Management: Biodiversity Act (NEMBA), 2014). Furthermore, trading of *P. cordata* in South Africa is not permitted due to the plant's high invasive potential. Despite these restrictions in trade and legislation, backyard trading may facilitate the spread of the species, a common problem in South Africa (Afonso et al., 2022; Geerts et al., 2017; Martin & Coetzee, 2011). Management strategies to control and reduce the spread of *P. cordata* in South Africa are therefore crucial. We suggest the development of a biological control program, since it is expected that the most efficient control would come from a biological control agent (Paterson et al., 2009). Based on the findings of this study, surveys for biological control agents should prioritize insects that damage *P. cordata*'s rhizomes because asexual reproduction via rhizomes is responsible for the spread of *P. cordata* in South Africa. Additionally, engaging with the horticultural industry and the public is critical (Novoa et al., 2018).

This study is a rare example of reproduction hampering a biological invasion. Incompatibility in angiosperms is one of the most successful anti‐selfing mechanisms present in plant reproductive biology (Khanduri et al., 2013). This anti‐selfing mechanism is meant to strengthen the species genetic diversity and fecundity; however, in an invasive setting, it seems to disadvantage *P. cordata*'s spread. Sexual reproduction in *P. cordata* is not present in invasive South African populations due to self‐incompatibility and illegitimate pollination of short‐morphed plants. Several studies have shown that the short‐morphed flowers from *P. cordata* have the greatest incompatibility system (Barrett, 1976; Gettys, 2005; Ornduff, 1966). Ornduff (1966) reported that an infinitesimal 5.3% of illegitimately pollinated short‐morphed *P. cordata* flowers studied produced seed‐bearing fruit. It is therefore unsurprising that 0% of illegitimately pollinated flowers from invasive populations of short‐morphed plants in this study produced seeds. This incompatibility disadvantage hampering *P. cordata* invasion in South Africa is an opportunity for management strategies to control the invasion while plants are only spreading asexually via rhizomes.

## AUTHOR CONTRIBUTIONS

**Sage Wansell:** Formal analysis (lead); investigation (lead); writing – original draft (lead). **Sjirk Geerts:** Conceptualization (equal); methodology (equal); supervision (supporting); writing – review and editing (equal). **Julie A. Coetzee:** Conceptualization (lead); funding acquisition (lead); methodology (equal); project administration (lead); supervision (lead); writing – review and editing (lead).

## Supporting information

Appendix

## Data Availability

The data supporting the findings presented in this study are accessible via Dryad digital repository: https://doi.org/10.5061/dryad.ht76hdrj0.
