# Supplementary material for: Where are the seeds? Lack of floral morphs prevent seed production by the tristylous Pontederia cordata in South Africa
Source: Ecol Evol. 2022 Oct 1;12(10):e9366. doi: 10.1002/ece3.9366 (PMC9526117; doi:10.1002/ece3.9366)
Supplement: Supplementary file 1 — Appendix [file ECE3-12-e9366-s001.docx]

*Supporting information*

**Table S1.** Factor coordinates and Eigenvalues from Principal Component Analysis of floral organs of *Pontederia cordata* in South Africa.

| **Flower organs:** | **Factor 1** | **Factor 2** | **Factor 3** | **Factor 4** | **Factor 5** | **Factor 6** |
| --- | --- | --- | --- | --- | --- | --- |
| Pistil | 0.62786 | -0.22303 | 0.74206 | 0.07075 | -0.01974 | 0.00290 |
| Petal length | 0.83219 | -0.27837 | -0.22609 | 0.05029 | -0.41324 | -0.07467 |
| Medium filament | 0.91596 | -0.03448 | -0.14864 | -0.05181 | 0.32217 | -0.17678 |
| Long filament | 0.94581 | -0.04510 | -0.18612 | -0.00544 | 0.10531 | 0.24010 |
| Medium anther | -0.31280 | -0.79955 | -0.10649 | 0.49040 | 0.10494 | 0.00523 |
| Long anther | -0.23632 | -0.82867 | -0.00470 | -0.50665 | 0.02373 | 0.01355 |
| **Eigenvalues** | 2.97398 | 1.456422 | 0.66986 | 0.50744 | 0.29760 | 0.09469 |
| **% Total variance** | **49.56631** | **24.27369** | **11.16440** | **8.45734** | **4.96002** | **0.57824** |

**Table S2.** Results of ANOVAs comparing *Pontederia cordata* floral organ morphology among populations in South Africa.

| Flower organ: | F-value _(8, 2151):_ | *P*-value |
| --- | --- | --- |
| Medium filament | 257.36 | <0.001 |
| Long filament | 262.83 | <0.001 |
| Medium anther | 27.03 | <0.001 |
| Long anther | 46.40 | <0.001 |
| Pistil | 21.84 | <0.001 |
| Petal length | 60.90 | <0.001 |

**Table S3.** Kruskal-Wallis ANOVA values testing for differences in insect visitation rates among different populations of invasive *Pontederia cordata*.

| Location: | Kruskal-Wallis test: | P-value: |
| --- | --- | --- |
| Pietermaritzburg, KwaZulu-Natal | H (3, N = 16) = 3.852897 | 0.2778 |
| Underberg, KwaZulu-Natal | H (5, N= 30) = 7.781782 | 0.1687 |
| Port Elizabeth, Eastern Cape | H (3, N = 26) = 1.781970 | 0.6189 |
| East London, Eastern Cape | H ( 4, N= 17) =3.313443 | 0.5068 |
| Port Alfred, Eastern Cape | H (3, N= 13) =0.2971271 | 0.9606 |


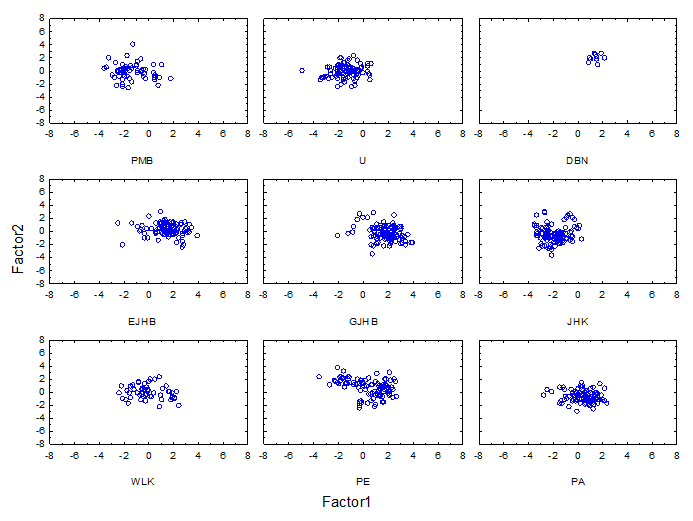


**Figure S1.** Principal Component Analysis of floral organs of *Pontederia cordata* from invasive populations in South Africa shown separately. PMB: Pietermaritzburg, KwaZulu-Natal. U: Underberg, KwaZulu-Natal. DBN: Durban, KwaZulu-Natal. EJHB: Emmarentia Botanical Gardens in Johannesburg. GJHB: Glenhazel, Johannesburg. JHK:Jonkershoek, Western Cape. WLK: Westlake, Western Cape. PE: Port Elizabeth, Eastern Cape. PA: Port Alfred, Eastern Cape.

| 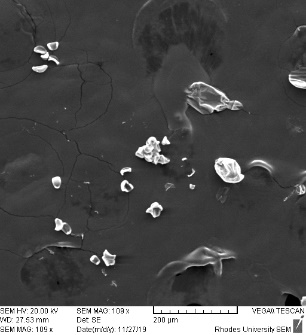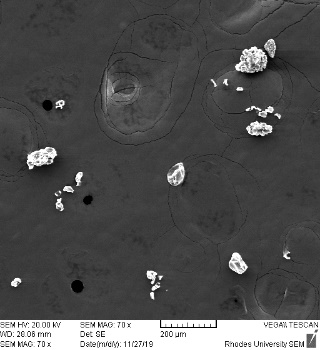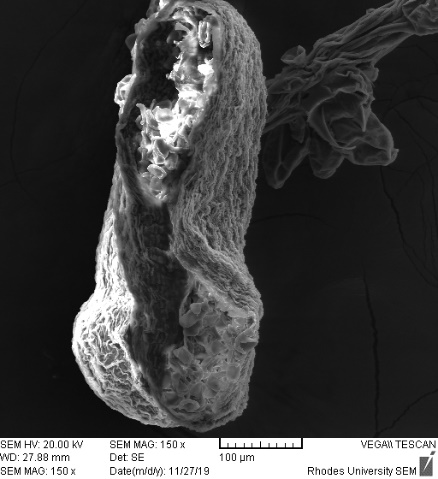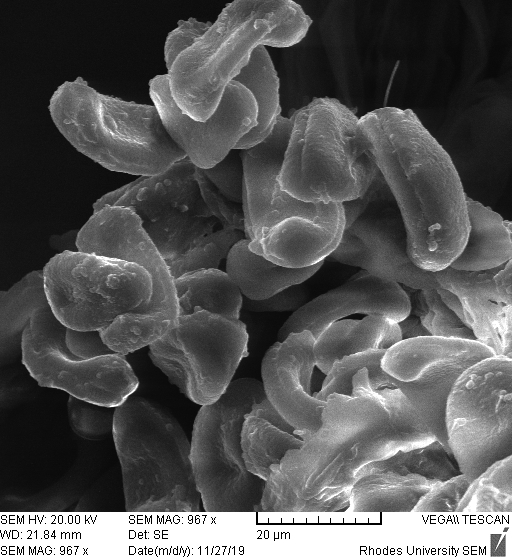  C  B  B  A  **200 µm**  **200 µm**  **100 µm**  **20 µm** |
| --- |

**Figure S2.** Scanning Electron Microscope images of the pollen grain morphology from flowers of *Pontederia cordata* in South Africa. A: pollen grains from a medium anther stored in alcohol. B: fresh pollen grains. C: pollen grains attached to an anther stored in alcohol.


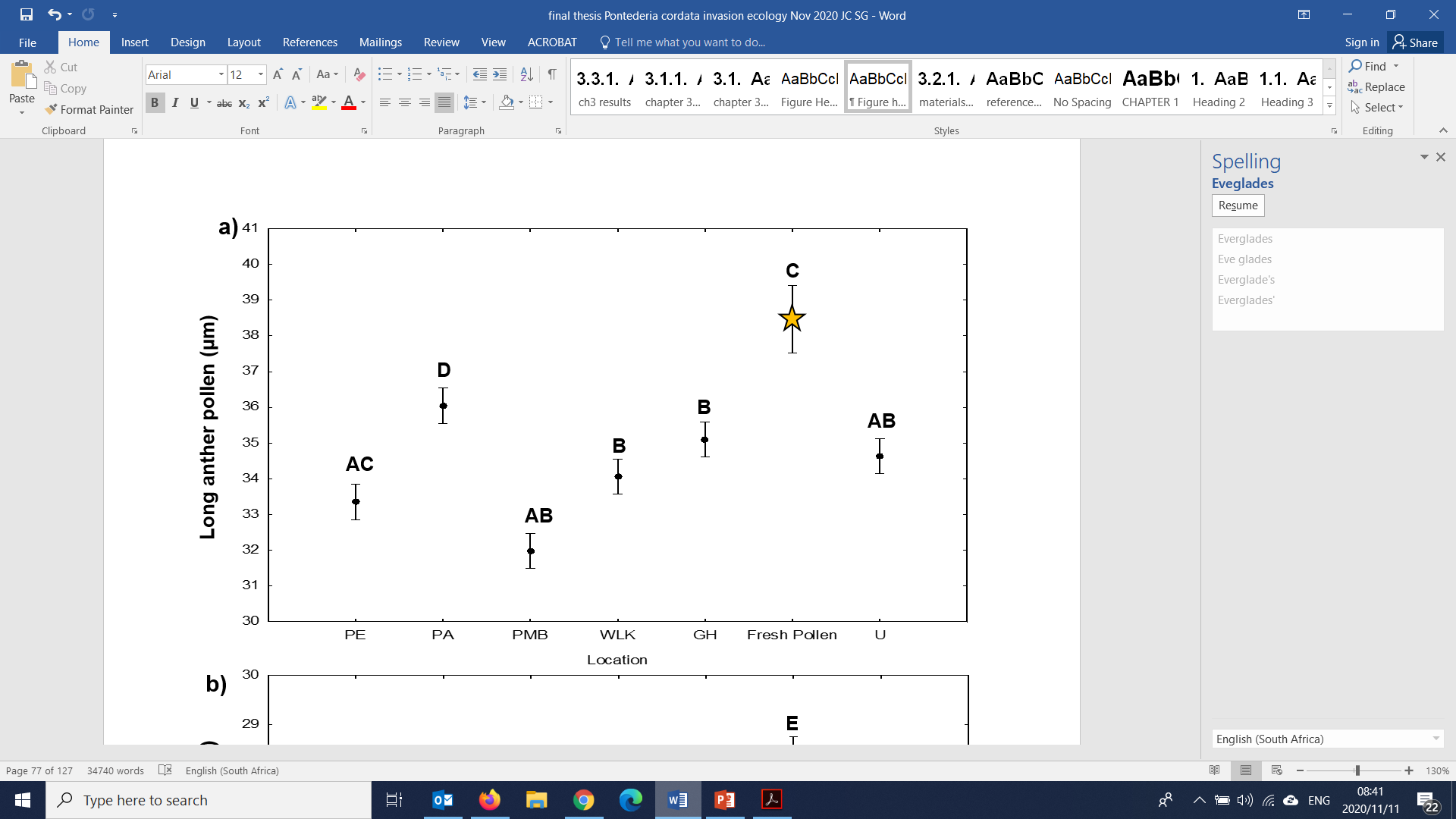

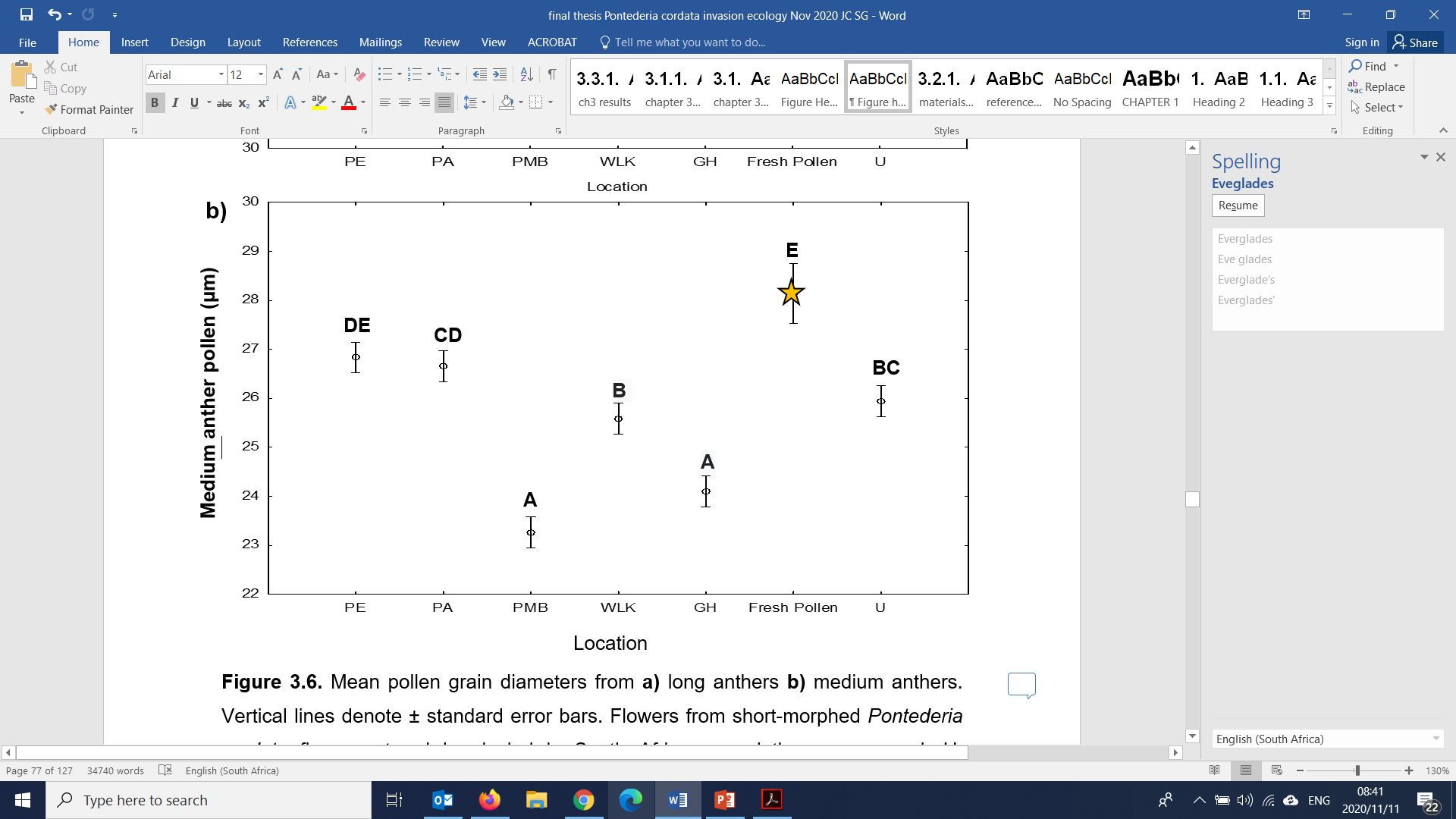


**Figure S3.** Mean pollen grain diameters from **a)** long anthers **b)** medium anthers. Vertical lines denote ± standard error bars. Flowers from short-morphed *Pontederia cordata* flowers stored in alcohol in South African populations were used. U: Underberg. PE: Port Elizabeth. PA: Port Alfred. PMB: Pietermaritzburg. WLK: Westlake. GH: Glenhazel. Fresh pollen from Hogsback indicate with star. Letters indicate population similarity using Tukey’s HSD test. **a)** *F* _6, 457_= 10.150, *P* < 0.0001. **b)** *F* _6, 457_= 20.518, *P*< 0.0001.
